# Adrenocortical Carcinomas: Molecular Pathogenesis, Treatment Options, and Emerging Immunotherapy and Targeted Therapy Approaches

**DOI:** 10.1093/oncolo/oyae029

**Published:** 2024-02-21

**Authors:** Divya Chukkalore, Kira MacDougall, Viraj Master, Mehmet Asim Bilen, Bassel Nazha

**Affiliations:** Department of Internal Medicine, Staten Island University Hospital, New York, NY, USA; Department of Hematology and Medical Oncology, the University of Oklahoma Medical Center, Oklahoma City, OK, USA; Department of Urology, Emory University School of Medicine, Atlanta, GA, USA; Winship Cancer Institute of Emory University, Atlanta, GA, USA; Winship Cancer Institute of Emory University, Atlanta, GA, USA; Department of Hematology and Medical Oncology, Emory University School of Medicine, Atlanta, GA, USA; Winship Cancer Institute of Emory University, Atlanta, GA, USA; Department of Hematology and Medical Oncology, Emory University School of Medicine, Atlanta, GA, USA

**Keywords:** adrenocortical carcinoma, adrenal cancer, targeted therapy, immunotherapy, therapeutic options

## Abstract

Adrenocortical carcinoma (ACC) is a rare and aggressive malignancy in the advanced setting with poor prognosis. This narrative review provides an overview of the epidemiology of ACC and its molecular pathogenesis with a summary of the main involved signaling pathways. We then provide an update on the clinical presentation, diagnosis, and current management strategies of both localized and metastatic disease from a multidisciplinary perspective. We highlight the debate around the use of mitotane in the adjuvant setting and review the use of combination chemotherapy with etoposide, doxorubicin, and cisplatin. The review also focuses on emerging data providing hope for the use of immune checkpoint inhibitors and targeted therapies in ACC with a summary of ongoing trials.

## Teaser

This review reports on the epidemiology of adrenocortical carcinoma and its molecular pathogenesis. An update on clinical presentation, diagnosis, and current management strategies is provided, as well as emerging data on the use of immune checkpoint inhibitors and targeted therapies.

**Implications for Practice**

Adrenocortical carcinoma (ACC) is a rare aggressive malignancy of the adrenal cortex with limited effective treatment options in advanced settings beyond conventional chemotherapy. This narrative review contributes to understanding the molecular basis of ACC, summarizes the current management approaches from a multidisciplinary perspective, and explores the advances in the use of immune checkpoint inhibitors and targeted therapies in this devastating disease.

## Introduction

Adrenocortical carcinoma (ACC) is a rare and aggressive tumor derived from the adrenal cortex, with an estimated annual incidence of 0.72 per million. ACC is also known to have a female preponderance and a bimodal age distribution, with peaks during childhood and in the fourth decade of life.^1-3^ A higher incidence of ACC has been noted in the southern parts of Brazil, particularly during childhood. This is believed to be due to a higher prevalence of the tumor protein p53 (TP53) germline mutation of the tumor suppressor gene allele R175H and R337H alleles.^2,4^ ACCs are classically associated with unfavorable outcomes: they have an estimated 5-year overall survival (OS) rate of <35% with a recurrence rate of as high as 70%-80%, although less so with lower stages.^5^ Physiologically, ACCs are classified as either functional or nonfunctional based on hormone production. Functional ACCs are associated with symptoms of excessively secreted hormones which may result in Cushing’s syndrome, virilization, or hyperaldosteronism. Nonfunctional tumors do not produce hormones and as a result present at a later stage. Approximately 1%-11% of tumors are incidentally diagnosed radiographically.^6,7^ Unfortunately, 25%-30% of overall patients with ACC have distant metastasis at initial disease presentation resulting in a dismal prognosis.^8^

The first-line therapeutic approach for localized ACC is complete surgical resection of the tumor with curative intent.^9^ Even with complete surgical resection, over half of the patients experience disease recurrence within 5 years, a risk that heavily depends on the initial disease stage and the surgical margin status after resection.^7^ Administration of mitotane alone or in combination with cytotoxic chemotherapy is considered in adjuvant settings to improve outcomes. Mitotane is currently the only approved adrenolytic agent but has limited efficacy with an objective response rate (ORR) of approximately 24% in advanced ACC.^9,10^ As this therapeutic strategy remains unsatisfactory, there is a critical need for new therapeutic options to improve survival rates and decrease recurrence risk. We provide a narrative review on the molecular basis of ACC, the clinical approach to treatment decisions from a multidisciplinary perspective, and the emerging data on the use of immune checkpoint inhibitors (ICI) and targeted therapies in the management of advanced ACC.

## Molecular Pathology

**Figure 1.**
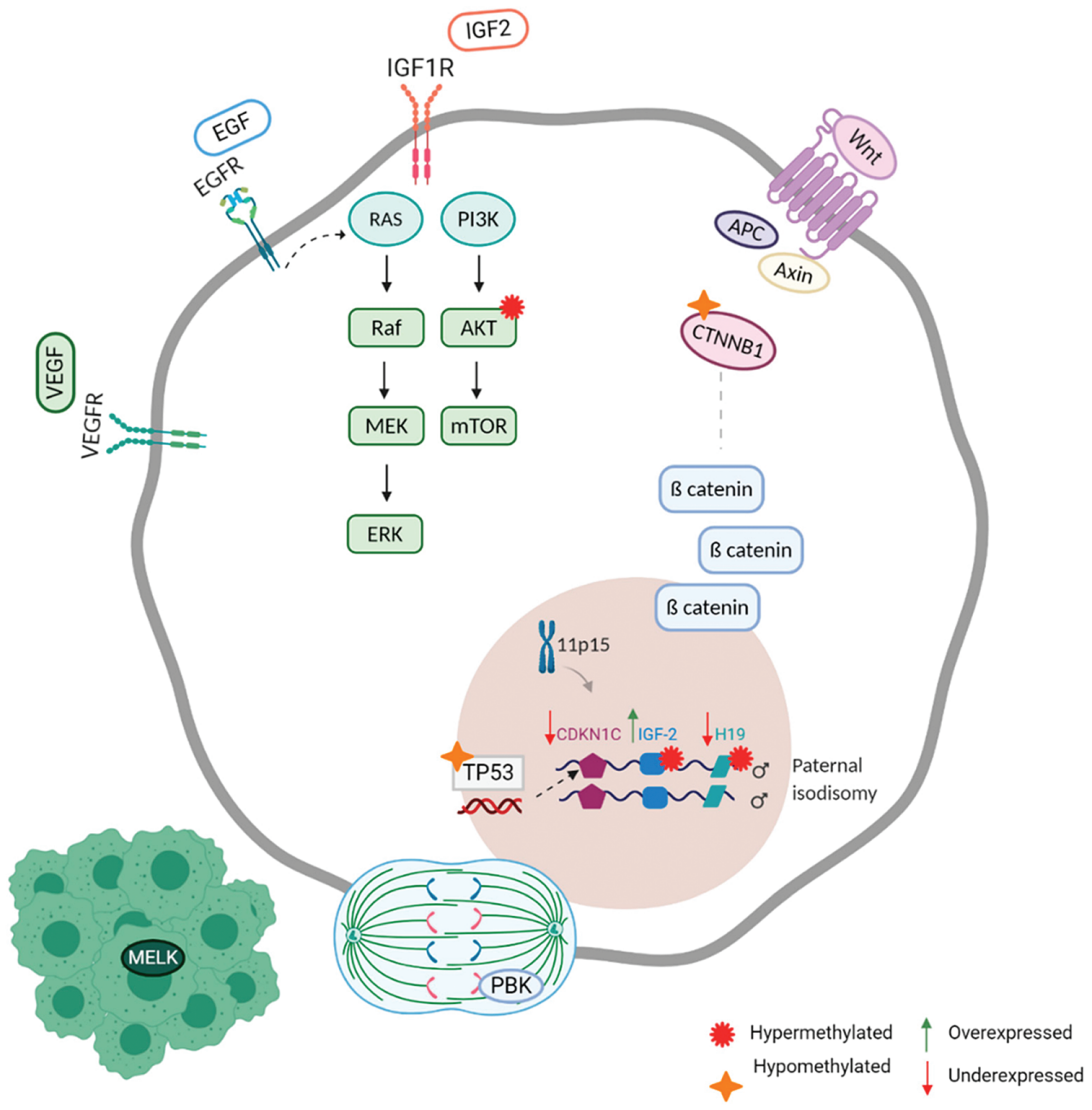
Molecular pathology and signaling pathways in ACC.

The molecular pathogenesis of ACC is marked by a multitude of oncogenic processes (Table 1).^19-21^ Many of these associated mutations lead to the dysregulation of the cyclic adenosine monophosphate (cAMP) signaling pathway and aberrant expression of growth factors, consequently activating the Wnt/β-catenin pathway (Figure 1).^18,22^

**Table 1.** Summary of the most common molecular mutations and pathways in ACC.

Type	Gene	Role
Tumor suppressor genes	TP53 [11]	Cycle arrest and repair damage; places cell in a state of senescence or induce apoptosis
	PRKAR1A[12]	DNA damage response and induction of proapoptotic signals
	MEN1 [13]	Histone modification and epigenetic gene regulation through several pathways; control cellular proliferation
Oncogenes	IGF-II [14,15]	Downregulation of transcription factors
Growth factors	VEGFR EGFR/TGF-α/TGF-β1/FGF-2/interleukins [16]	Tyrosine-kinase-coupled receptor-mediated cellular proliferation, survival, angiogenesis, apoptosis resistance, and metastasis
DNA methylation and epigenetics	Various [17]	Silencing of tumor suppressor genes and/or activation of oncogenes
Signaling molecules	Wnt/B-catenin pathway [18]	Activation of genes of pro-growth and pro-proliferation genes

### Tumor Suppression Genes

#### *TP53* gene

TP53, located within 17p13, plays a pivotal role in regulating various cellular processes, including cellular proliferation, DNA repair, and apoptosis.^11^ Approximately 70% of germline mutations in TP53 are associated with Li-Fraumeni syndrome, which predisposes individuals to various cancers, including ACC.^11^ Southern Brazil exhibits a higher incidence of adrenocortical tumors, especially in patients in pediatric care, linked to a higher prevalence of TP53 germline mutations.^23^ In sporadic ACC cases, somatic TP53 mutations are found in approximately 25% of instances.^24,25^

#### *PRKAR1A* gene

The PRKAR1A gene, located on the 17q22-24 chromosomal locus, is crucial in the cAMP signaling pathway and is linked to Carney complex, a rare hereditary syndrome.^12^ This syndrome affects approximately 700 individuals worldwide and is characterized by several distinctive features, including somatotroph pituitary adenomas, thyroid adenomas, primary pigmented nodular adrenal disease, calcifying Sertoli cell tumors, lentigines, and myxomas.^12^

#### *MEN1* Gene

Mutations in the MEN1 gene are associated with an increased risk of developing endocrine neoplasms due to the loss of normal menin function which can result in ACC development. However, MEN1 mutations are not as frequently associated with ACC as they are with other types of tumors, such as pancreatic neuroendocrine tumors.^25^

### Oncogenes

#### Insulin Growth Factor II

Insulin growth factor II (IGF-II) is a crucial fetal growth factor involved in adrenal cortex development and is present in a substantial percentage of ACC cases.^14,25^ IGF-II is regulated by the 11p15 chromosome locus, which includes the IGF-II coding region, H19, and CDKN1C1, among others.^26^ Dysregulation of this locus leads to upregulated IGF-II expression and is associated with Beckwith-Wiedemann syndrome, an adrenal phenotypic disorder.

### Growth Factors

Numerous growth factors and cytokines have been implicated in ACC. These include transforming growth factor-α (TGF-α), TGF-β1, vascular endothelial growth factor (VEGF), fibroblast growth factor (FGF-2), and various interleukins.^25,27^ These factors exert their effects through tyrosine-kinase-coupled receptors, influencing processes such as cellular proliferation, survival, angiogenesis, resistance to apoptosis, and the potential for metastasis.^2,28^

### Chromosomal Changes

Chromosomal alterations can be either a loss or gain and have been associated with the pathogenesis of ACC development. Chromosomal losses were reported to occur at the following loci: 1p, 21, 11q, 17p, 22p, and 22q1, whereas chromosomal gains occur at 4q, 4p16, 5p15, 5q12-13, 5q32-qter, 9q34, 12q13, and 19p.^15,25^

### TERT Promoter Mutation

Activation of telomerase is observed in some adrenocortical cancers. The TERT promoter mutation C228T disrupts the regular control of the TERT gene, resulting in increased telomerase expression. This allows ACC cells to evade typical mechanisms limiting cell division.^29^ A study by Zheng et al comprehensively analyzed 91 ACC specimens, and demonstrated that 73% of tumors had shorter telomeres, particularly in whole‐genome duplication cases, possibly indicating a compensatory role for TERT in telomere maintenance.^30,^^31^

### DNA Mismatch Repair Genes

Aberrations in DNA mismatch repair (MMR) genes, leading to microsatellite instability, are implicated in a subset of ACC cases.^32^ Lynch syndrome, caused by germline variants in MMR genes (MLH1, MSH2, MSH6, PMS2, and EPCAM), is associated with ACC development.^33^ MMR-deficient tumors possess a high somatic mutation burden resulting in increased tumor-specific T-cell responses making them potential targets for immunotherapy programmed death-ligand 1 (PD-L1).^34^

### Methylation and Epigenetics

DNA epigenetic modifications in ACC include DNA methylation, messenger RNA expression changes, and microRNA expression changes.^17,35^ DNA methylation is a common epigenetic alteration in ACC, resulting in the silencing of tumor suppressor genes and activation of oncogenes.^27^ CpG island methylation has been identified as an independent prognostic marker of survival in ACC.^35^

### Wnt/B-Catenin Pathway

Oncogenic aberrations associated with ACC consist of the activation of the Wnt/β-catenin pathway.^28^ A study investigated whether Wnt pathway activation is involved in adrenocortical tumorigenesis in which 21 of 39 tumors (54%) had an alteration in β-catenin.^18^ This study suggested that the activation of the Wnt signaling pathway is the most common abnormality in adrenocortical tumorigenesis.^18^ In sporadic adrenocortical adenomas and ACC, the most common defect is due to genetic alterations in exon 3 of the somatic β-catenin gene (*CTNNB1*).^36^

## Clinical Presentation

ACC has a variable clinical presentation. Around 30% have nonspecific symptoms such as abdominal pain and/or fullness, flank pain, and early satiety. Diagnoses made following work up of adrenal mass incidentally found on imaging account for 20%-30% of cases.^20,37^ Hormonal excess is the most common presenting feature in approximately 40%-60% of patients which could be due to hypercortisolism or hyperandrogenism. ACC that secretes cortisol may cause patients to present with diabetes mellitus, osteoporosis, facial plethora, muscle atrophy, secondary hypertension, and/or hypokalemia.^20,37^ Conversely, aldosterone over-secretion that is manifested by hypertension and hypokalemia is rare in ACC.^20,37^ Patients may present with virilization, hirsutism, male pattern hair loss, and menstrual abnormalities. A few patients can present with clinical symptoms correlated to estrogen overproduction such as testicular atrophy and gynecomastia.^37^

## Diagnosis and Staging

It is judicious to maintain a wide differential diagnosis when evaluating adrenal masses suspicious for ACC, as those could be benign (adenomas), metastatic (eg, from non–small cell lung cancer), or pheochromocytomas, among other potential etiologies. ACC is diagnosed upon careful clinical, biochemical, radiological, and histological assessment. The authors do not recommend fine-needle aspirations of the adrenal mass due to the risk of malignant cell seeding.^38^

Imaging studies such as computerized tomography, magnetic resonance imaging, and 18F-fluorodeoxyglucose positron emission tomography (FDG-PET) are commonly used to diagnose and differentiate benign from malignant lesions.^3^ The average size of ACC at diagnosis is estimated to be 10-13 cm with only 3% of the cases presenting with a tumor size of 4 cm or less.^5^ As such, clinical suspicion of a malignant process like ACC is heightened for tumors larger than 5 cm. Once the diagnosis is made, FDG-PET can be particularly useful in discerning bony metastatic disease in ACC.^38^

After a surgical specimen is obtained, the Weiss score (0-9) is considered the gold standard tool for differentiating benign from malignant adrenocortical lesions. The score consists of 9 histopathologic criteria: eosinophilic cytoplasm in more than 75% of tumor cells, a patternless diffuse architecture, atypical mitoses, necrosis, nuclear atypia, mitotic index above 5 per 50 high-power fields, sinusoidal, venous, and capsular invasion.^39^ A diagnosis of ACC is defined by a score of ≥3, whereas scores between 0 and 2 correlate to an adrenal adenoma.^39^ When available, the authors recommend a second pathology opinion from a dedicated endocrine or genitourinary pathologist given the rarity of ACC and the emphasis placed on low-grade versus high-grade disease.

The initial staging system for ACC follows the American Joint Committee on Cancer (AJCC) TNM staging, with T3 or any nodal involvement being Stage III or higher (Table 2).

**Table 2.** Staging system for ACC per the AJCC 2017.

Stage	TNM
I	T1, N0, M0 Tumor ≤ 5 cm
II	T2, N0, M0 Tumor > 5 cm
III	T1-2, N1, M0 or T3-4, N0-1, M0
IV	T1-4, N0-1,M1

## Management of Localized Disease

### Surgical Resection

The management of ACC poses several unique challenges as it often involves oncologic and endocrine considerations. The mainstay of treatment for ACC is surgical resection. For patients with stage I or II and some stage III, radical surgical resection is the only potentially curative option.^2^ Patients should undergo a hormonal assessment prior to excision, to determine whether the tumor secretes cortisol, and whether they are at risk for postoperative adrenal insufficiency and should receive replacement therapy.^10^ Open adrenalectomy with surgical excision of lymph nodes is recommended by the National Comprehensive Cancer Network (NCCN), American Association of Clinical Endocrinologists, and the Association of Endocrine Surgeons (AAES) because it minimizes the risk of peritoneal spread and allows for resection of adjacent structures when necessary.^40^ Further, the authors recommend a high degree of diligence during surgery to curtail the chance of capsular breach as ACC membranes tend to be thin.

For complete resection, it may be necessary to remove surrounding organs such as the ipsilateral kidney, pancreas, spleen, liver, and/or diaphragm. Achieving negative surgical margins is key to decrease the risk of recurrence.

### Neoadjuvant Therapy

Due to a lack of randomized trials in this space, the role of neoadjuvant chemotherapy for borderline resectable ACC is not well defined. One single-center retrospective review of 53 patients suggested favorable outcomes with neoadjuvant cisplatin-based chemotherapy, but prospective studies are needed before definitive conclusions can be drawn.^41^ For patients who are surgical candidates, the authors recommend undergoing surgical resection first rather than neoadjuvant chemotherapy.

### Adjuvant Therapy

A patient’s risk of recurrence after surgery determines the need for adjuvant therapy. Tumor stage, completeness of resection, and proliferation rate are the 3 key prognostic factors that influence recurrence risk.^42^ Historically, oral mitotane has been the agent of choice for adjuvant therapy yet this remains a subject of significant controversy.^43^ Mitotane has a cytotoxic effect on adrenal tissue and inhibits steroidogenesis, making it an effective agent in this disease. A systematic review and meta-analysis looked at 1249 patients with ACC receiving adjuvant mitotane. They suggested that adjuvant mitotane significantly decreases the recurrence rate and mortality after resection of ACC in patients without distant metastasis.^44^ More recently, a randomized trial of 91 patients with low-risk ACC compared adjuvant mitotane for a minimum of 2 years to observation alone. The trial found that patients with a low or intermediate risk of recurrence after surgery do not benefit from adjuvant mitotane, reducing enthusiasm regarding this adjuvant option.^45^ Therefore, patients with low- or intermediate-risk disease can avoid the side effects of mitotane, which can include nausea, vomiting, diarrhea, anorexia, depression, dizziness, vertigo, etc. Serum mitotane levels should be monitored closely, as they correlate with side effects.^46^

The authors consider adjuvant mitotane in patients with high-risk disease, including those with positive surgical margins, a ruptured capsule, large size (no specific cutoff is offered in the available treatment guidelines), and/or a high tumor grade (Ki-67 staining of >10%).^47^ A target mitotane level of 14-20 µg/mL should be reached. The authors check mitotane levels for months; it can take several months and often requires close follow-up with endocrinology.^48^ Replacement doses of corticosteroids are frequently required due to the adrenolytic effects of mitotane and could be needed over the patient’s lifetime. For patients with very high-risk diseases, including those with Ki-67 staining ≥20%, extensive vascular invasion, or vena cava thrombus, the authors discuss and often favor off-label platinum-based chemotherapy in addition to adjuvant mitotane, although the benefits of chemotherapy in this setting have yet to be demonstrated.^8^ This will be answered in the ongoing Adiuvo-2 trial (NCT03583710), a phase III trial of mitotane with or without cisplatin and etoposide after surgical resections in patients with high-risk ACC. Lastly, adjuvant external beam radiation therapy to the surgical bed is a treatment option though this is not routine practice in the authors’ multidisciplinary practice as ACC is considered a radioresistant cancer. Also, questions remain regarding whether this reduces the risk of metastatic recurrence or improves OS.

## Management of Metastatic Disease

There is currently no curative intent systemic therapy for recurrent or metastatic ACC. In a series of 113 patients with locally recurrent disease, those who underwent complete surgical resection of known disease sites were found to have improved survival compared to those who did not.^49^ Surgical resection may also be considered in those who have oligometastatic disease to the liver or lung when all known sites of disease can be resected.^50^

For patients with metastatic disease, mitotane monotherapy has been studied but the ORRs are low, at 10%-30%, and therefore combined therapy is generally recommended.^51^ Of note, mitotane is the only FDA-approved therapy specifically for ACC to date. A phase II study of 72 patients with ACC demonstrated an ORR of 49% in patients who received etoposide, doxorubicin, and cisplatin (EDP) in combination with mitotane.^52^ A larger study of 304 patients with advanced ACC, The First International Randomized Trial in Locally Advanced and Metastatic Adrenocortical Carcinoma Treatment (FIRM-ACT), randomly assigned patients to mitotane plus EDP or mitotane plus streptozotocin. The study found that when compared to mitotane plus streptozotocin, EDP plus mitotane resulted in an improvement in both response rate (23.2% vs 9%, *P* < .001) and progression-free survival (PFS; 5.0 months vs 2.1 months; hazard ratio (HR), 0.55; 95% CI, 0.43-0.69, *P* < .001), but not OS (14.8 months vs 12.0 months, HR, 0.79; 95% CI, 0.61-1.02; *P* = .07).^53^ The median number of received cycles was 4 for patients on the EDP plus mitotane arm. This trial established EDP plus or minus mitotane every 28 days as first-line standard of care for metastatic ACC.

No standard or consistent second-line systemic therapy options exist post-progression on mitotane ± chemotherapy, a setting with very poor prognosis. This led to the exploration of ICI in ACC. The ICI studies are marked by variable albeit low ORRs in an unselected treatment approach (Table 3). Several small phase II trials have investigated pembrolizumab, an anti-PD-1 antibody in patients with advanced ACC. One phase II trial (NCT02721732) consisted of 14 patients in which 5 of the 14 patients were alive and progression free at 27 weeks (nonprogression rate at 27 weeks was 36% [95% CI, 13%-65%]).^54^ Of the 14 patients, 2 had a partial response (PR), 7 had stable disease, and 5 had progression of disease. The ORR was 14% (95% CI, l2%-43%). Of the patients with stable disease, 6 had disease stabilization lasting more than 4 months, a novel and encouraging finding not seen previously with platinum-based chemotherapy. The authors believe that this supports that a subset of patients with ACC have indolent disease kinetics, an observation previously reported by Assié et al^55^ based on distinct molecular alterations. In another phase II trial (NCT02673333), 39 patients with advanced ACC were monitored for a primary endpoint of ORR to pembrolizumab. The ORR was 23% (9 patients; 95% CI, 11%-39%), disease control rate of 52% (16 patients; 95% CI, 33%-69%), with a median PFS of 2.1 months (95% CI, 2.0-10.7 months), and median OS of 24.9 months (95% CI, 4.2 months to not reached).^30^ A third phase II study of pembrolizumab in advanced ACC found an ORR of 15% and a clinical benefit rate of 54%.^54^ Single-agent pembrolizumab is now listed in the NCCN guidelines as a treatment option in advanced ACC, including the first-line setting. Pembrolizumab would be a treatment of choice in patients who are unable to tolerate EDP chemotherapy or who are felt to have a low symptom burden or nonrapid disease kinetics of their metastatic ACC. Considering the steroid-related immunosuppression that is likely to limit the effectiveness of immunotherapy in ACC, this approach carries the risk of inadequate treatment.^56^ The authors believe that combination chemotherapy should be given whenever possible.

**Table 3.** Published and ongoing ICI and targeted therapies for advanced or metastatic ACC.

Drug	Target	Study phase	Patients	Results	Ref.
Avelumab	PD-1	I	50	ORR: 6% OS: 10.6 months PFS: 2.6	NCT01772004
Pembrolizumab	PD-1	II	39	ORR: 23.1% (95% CI, 11.1-39.3) PFS: 2.1 mo (95% CI, 2.0-10.7) OS: 24.9 mo (95% CI, 4.2-not reached) 2-year OS rate was 50% (95% CI, 36-69%)	NCT02673333
Pembrolizumab	PD-1	II	16	Median follow-up was 27.3 months ORR was 20.0% (95% CI, 6.8-40.7) Median PFS: 4.1 (95% CI, 3.1-5.1) OFS: 11.3 (95% CI 5.5-17.1) months, respectively	NCT02721732
Pembrolizumab	PD-1	II	Not yet recruiting	—	NCT05563467
Ipilimumab and radiotherapy	CTLA-4	I/II	Active, nonrecruiting	—	NCT02239900
Nivolumab	PD-1	II	Terminated	—	NCT02720484
Nivolumab and ipilimumab	PD-1/CTLA-4	II	Recruiting	—	NCT03333616
Pembrolizumab and Relacorilant	PD-1/GR antagonist	Ib	Recruiting	—	NCT04373265
Camrelizumab and Apatinib	PD-1 VEGF	II	Recruiting		NCT04318730
Nivolumab and Ipilimumab	PD-1 CTLA-4	II	Recruiting	—	NCT02834013
Pembrolizumab and Lenvatinib	PD-1 VEGF	II	Recruiting	—	NCT05036434
Dovitinib	FGFR	II	17	ORR: 0% PFS: 1.8 months Stable disease (>6 months): 23%	NCT01514526
Cabozantinib	c-MET, VEGF, AXL, and RET	II	Recruiting	—	NCT03612232
Linsitinib	IGF-1R and the insulin receptor	III	139	No improvement in PFS or OS	NCT00924989
Cixutumumab and Temsirolimus	IGF-1R and mTOR	I	26	Stable disease > 6 months: 42%	NCT00678769
Sunitinib	VEGFR1, VEGFR2, c-KIT, Fms-like tyrosine kinase 3, PDGFR	II	38	PFS: 2.8 months OS: 5.4 months	NCT00453895
Nevanimibe	Acyl-coenzyme A:cholesterol *O*-acyltransferase 1	I	63	Stable disease at 2 months: 27% Stable disease at 4 months: 8%	NCT01898715
Axitinib	VEGF	II	13	PFS: 5.48 months OS: 13.7 months	NCT01255137

Nivolumab, a PD-1 inhibitor, was also evaluated as monotherapy in a phase II trial (NCT02720484) in ACC. The primary endpoint was ORR. Ten patients with advanced ACC who were previously treated with or declined platinum-based first-line therapies were included and received nivolumab 240 mg i.v. every 2 weeks.^57^ The median PFS was 1.8 months, a finding that reflects the aggressive disease course beyond first-line settings. The best response observed in this trial was 1 of 10 patients with an unconfirmed PR and 2 of 10 patients with stable disease.^57^ However, given the lack of confirmed PR in the first 10 patients, the trial was terminated.

Avelumab, a PD-L1 inhibitor, was evaluated in a phase Ib clinical trial (NCT01772004) in patients with metastatic ACC who progressed after the first-line platinum-based therapy. This trial consisted of 50 patients who were treated with avelumab 10 mg/kg regardless of concurrent mitotane therapy. An ORR of 6% was observed, with 3 patients displaying PR. The median PFS and OS were 2.6 and 10.6 months, respectively.^58^ Adverse effects reported were mild and well tolerated.

Approximately 50% of ACCs generate glucocorticoids (GC), and hypercortisolism is associated with reduced survival rates in patients with ACC. Blocking the GC receptor (GR) holds the potential for enhancing immune-related gene expression, which could stimulate an immune response against the tumors with GC excess. To investigate this hypothesis, a phase Ib clinical trial (NCT04373265) is assessing the combined use of relacorilant a nonsteroidal anti-GC and pembrolizumab in patients with advanced ACC and hypercortisolism.^59^

The realm of targeted therapy for ACC is still in its early stages, and there are currently no approved targeted therapies designed specifically for ACC. Further, there are no identifiable biomarkers yet that can reliably predict their effectiveness. Dovitnib, an inhibitor of fibroblast growth factor receptor (FGFR), was studied in a phase II trial of 17 patients in the first-line metastatic or locally advanced setting. No objective responses were observed, the median PFS was 1.8 months (95% CI, 1.35-2.25), and 23% of patients achieved stable disease at 6 months.^60^ Numerous studies are looking at targeting VEGF, either alone or in combination with ICI. The VEGF inhibitor axitinib was investigated in a phase II trial of 13 patients with metastatic ACC. The median PFS and OS were 5.48 months and 13.7 months, respectively.^61^ Cabozantinib, an inhibitor of c-MET, VEGF, AXL, and RET, is another agent currently under investigation. A retrospective cohort study of 16 patients with progressive ACC after mitotane (with the exception of one patient) treated with cabozantinib, resulted in 3 PRs and 5 cases of stable disease for 4 months or longer.^62^ The CaboACC phase II clinical trial (NCT03612232) is currently underway to further investigate this agent in the advanced setting. Bedrose et al described the combination of lenvatinib (a multikinase VEGF inhibitor) with pembrolizumab in a small retrospective case series as salvage therapy in advanced ACC in heavily pretreated patients.^63^ The combination was reported to be well tolerated with no severe toxicities. Of the 8 enrolled patients, 2 (25%) had PR and 1 (12.5%) had stable disease, findings that are proof of concept for a potential role for ICI/anti-VEGF in this setting. The Accomplish phase II clinical trial (NCT05036434) is currently planned to use the combination of pembrolizumab and lenvatinib in advanced ACC. Sunitinib was studied in a phase II trial of 35 patients with refractory ACC. The median PFS and OS were 2.8 and 5.4 months, respectively.^64^

Other novel targeted agents are also being explored in the metastatic setting. In a phase I study, a combination of cixutumumab, an inhibitor of IGF-1R (insulin-like growth factor 1 receptor), and temsirolimus, an inhibitor of mTOR (mammalian target of rapamycin), demonstrated extended periods of stable disease. After more than 6 months, 11 out of 26 patients (42%) showed sustained disease stability.^65^ However, it is essential to consider the potential patient selection bias in this trial, as enrolling patients with low-volume or slow-growing diseases may have influenced these outcomes. Therefore, a randomized trial is needed to provide a more conclusive assessment. In a separate trial, Fassnacht et al^66^ conducted a phase III trial involving linsitinib, an inhibitor targeting both IGF-1R and the insulin receptor, compared to a placebo in patients with locally advanced or metastatic ACC. Unfortunately, the study did not reveal any significant improvements in either PFS or OS compared to the placebo group. Another novel agent studied in this setting is nevanimibe HCl, which is a selective inhibitor of acyl-coenzyme A:cholesterol *O*-acyltransferase 1. Nevanimibe was shown to lead to apoptosis of the adrenocortical cells in dogs by increasing free cholesterol, leading to endoplasmic reticulum stress and the unfolded protein response.^67^ Of the 48 patients who were included in the phase I study of nevanimibe, no patients experienced a complete or PR, but 13 patients (27%) had stable disease at 2 months and 4 (8%) and stable disease at 4 months.^68^

Next-generation testing (NGS) should be considered for patients with ACC to identify actionable mutations that may allow enrollment to molecularly selected basket clinical trials under an investigative approach. Indeed, available genomic landscape studies in ACC indicate a dominance of p53 mutations (present in over half of patients) yet with a variety of mutations with therapies currently in development in other cancers.^69,70^ For instance, around 20% of patients with ACC have CTNNB1 mutations, which promotes Wnt signaling. This pathway has been targeted in pancreatic cancer, ovarian cancer, and melanoma, among other malignancies. Tissues-based mutations in primary (newly diagnosed) and recurrent or metastatic samples are similar,^71^ suggesting that either sites of tissue is acceptable to obtain genomic studies. Although the availability of tissue for NGS is often a challenge, circulating tumor DNA (ctDNA) NGS of ACC is feasible with a study 80% detection rate of at least one somatic alteration in 120 tested patients.^72^

## Prognosis

ACC is an aggressive malignancy. A French study of 253 patients found that the 5-year actuarial survival rates were 38% for the overall population, 66% for stage I, 58% for stage II, 24% for stage III, and 0% for stage IV.^2^ For early-stage tumors, despite complete tumor resection, the rate of local recurrence ranges between 19% and 34%, based on the tumor stage.^2,20^ Factors associated with a worse prognosis include higher tumor stage at diagnosis, larger tumor sizes, older age, and hypercortisolism. Important prognostic factors that predict possible recurrence include hypercortisolism, higher Ki-67 index, larger tumor sizes, and advanced stages with incomplete surgical resection.^73^

## Conclusions

ACC are rare and aggressive malignancies with limited treatment options in the advanced settings. Surgery remains the only potentially curative treatment option. The role of adjuvant mitotane continues to be debated and can be offered for patients at a high risk of disease recurrence. EDP chemotherapy plus or minus mitotane is the standard frontline treatment approach for eligible patients with advanced disease. The recent data on durable responses with ICI in the form of stable disease is a significant advance in the field and offers hope. Studies with targeted therapies along with those combining ICI and VEGF inhibitors are underway and could inform the future care of these patients. Next-generation sequencing to facilitate participation in molecular selected clinical trials is encouraged for all patients facing this rare and aggressive cancer.

## Data Availability

No new data were generated or analyzed in support of this research.
